# The Department of Modern Nursing

**Published:** 1915-02-27

**Authors:** 


### THE DEPARTMENT OF MODERN NURSING.*

### IX. Guy's Hospital.

### THE PRELIMINARY TRAINING SCHOOL.

The benefit and result of a Preliminary Training School have been amply tested by the prevailing system at Guy's Hospital. When the "Henriette Raphael" Home for Nurses (opened in 1902) was built, its plans were made to include a "suite of apartments" necessary for the housing and instruction of sixteen pupil probationers. The pupils are entirely under the supervision of two sisters, whose sole occupation is their instruction, and they occupy a separate wing in the nurses' home, in which their class-room and kitchen is also situated. In the kitchen they are taught sick-room cookery by a qualified lady cook.

The fee for the course, including board, residence, tuition, and practical work, is six guineas, which must be paid in advance to the matron of the hospital; and the probationer has also to provide herself with indoor uniform and to pay for her personal laundry. The matron may at any time terminate the engagement of a preliminary probationer, and in such case the due proportion of the six guineas paid for training will be refunded. At the end of the course it rests with the matron to determine whether the probationer shall be allowed to continue her training. No separate certificate will be given for the course in the Preliminary Nursing School.

The plan of the pupils' work for each day is as follows:

#### Morning Routine.

8 to 10.30 a.m.—Housework. This comprises the care of all the nurses' rooms, the cleanliness of which the pupils are responsible for in every detail except scrubbing floors and cleaning windows; the beds in these rooms are made by them, and the taps and fixed basins cleaned. The nurses' sick-room on the floor immediately above their wing is swept and dusted and all brasses cleaned, the doctor's table is washed, fresh hot water is placed thereon, and the whole room is treated as a miniature ward, to be prepared for the medical officer's morning round as in the wards. Each pupil takes her turn at this work. The kitchen is cleaned at this hour, tables are scrubbed, cooking utensils cleaned, and lamps polished, and the pupils are entirely responsible for its order in every detail.

10.30 to 12.15.—Half the pupils attend class and the others cook and prepare dinner for any sick-room nurses, under the supervision of the sister.

Those at class do practical elementary nursing, such as the preparation of enemata, poultices, scrubbing of mackintoshes, etc.

12.15 p.m.—Pupils have dinner in their separate dining-room.

1 to 2 p.m.—Study in class-room on a given subject.

#### Afternoon Routine.

2 p.m.—All pupils are off duty, half from 2 to 3, and the others from 2 to 5.30 alternate days.

3.5 p.m.—According to the day the instruction for this hour is:

Monday and Tuesday.—Cooking, demonstrative and practical.

If there are any sick nurses, two or more pupils prepare and take up tea to the sick-room.

4 p.m.—Wednesday and Thursday.—Class on the making of dressings; how they are used; to what cases applied.

Friday.—The whole supply of bed-linen for the nurses' home comes from the laundry and is counted and sorted—torn or old articles being picked out—under the supervision of the dormitory sister. If there is any time to spare after this is done, dressings are proceeded with and mending of house linen is done.

Saturday.—Practical class in the nurses' sick-room, with a dummy, on bed-making in all its varieties—*e.g.*, patient coming back from operating theatre, accident admission, patient suffering from heart trouble. At these classes the uses and positions of sandbags, wedge pillows, hot bottles, and cradles are taught, also the giving and cleansing of bed-pans. After this class the use of the telephone is taught, and receiving and giving messages dealt with.

On the last Saturday, instead of bed-making, etc., a special class is given on the preparation of hypodermic injections and how to administer them; also names of simple instruments and their uses, the taking of swabs from throats for cultivation, and lotion-making.

#### Evening Routine.

5.30 to 6.45 p.m.—Every evening except Saturday a class is given on one of the following subjects:—Anatomy, hygiene, physiology, infant feeding, sepsis, and asepsis. The lecture just received in rough notes is copied out and the pupils are left to read one of the subjects, strict silence being observed.

Saturday.—There is no class. New uniform caps are made up and mending of clothes and uniform done, and during this time one of the sisters usually talks on ethical points, hospital etiquette, and the various points of interest socially in hospital, such as the use of the League.

Sunday.—Necessary work is done in the morning. Half the pupils go off duty from 10 a.m. to 9.30 p.m. The others are on duty and prepare sick-room meals.

Such is the routine of the Guy's pupil. Examinations in anatomy, physiology, and hygiene are held by an appointed member of the medical staff, and the cookery examination is conducted by Mr. Herman Senn. All three examinations must be passed, otherwise the pupil is not considered eligible for entrance into the wards.

In addition to all this, before accepted probationers enter the hospital, the operating theatres are

* Previous articles: May 16 and 30, June 13, July 11 and 25, August 8, 1914, and January 16 and 30, 1915.

inspected, the sterilisers are shown, and the theory of sterilisation of dressings is explained. The laundry is also shown and the methods of ward washing explained. The surgery and out-patient department are kept supplied with padded splints and cut lint by the school, and the successful results of cooking classes go to the wards for the patients whose diet includes those dishes and whose appetites need tempting.

### Trial Period in the Wards.

At the end of six weeks the pupils are drafted to the wards according to their suitability and their position on the pass list. During their first month in the wards the probationers are examined by the medical officer as to their physical fitness for the profession, and a dentist is also seen. If they are found strong enough and have earned two good reports by working for two months in different wards, from the sisters of those wards, they are required to sign an agreement to remain for three years' training. Having successfully passed through her six weeks in the training school and her two months' trial work in the wards—in some cases a third month's trial is given, at the discretion of the matron—the probationer really begins her three years' training in earnest, and receives an order for a fresh supply of indoor uniform. Outdoor uniform is also given at this time. The following statements give details of the succeeding events which form the routine of the three years:

### Probationers' Course.

On completion of her course in the Preliminary Nursing School, an applicant between the ages of twenty-three and thirty-two, and not less than 5 feet 1½ inch in height, may be received into the wards to be trained in medical and surgical nursing for the full period of three years. If accepted, she will be paid at the rate of £8 for the first year, £12 for the second year, and £18 for the third year (subject to the conditions specified in Regulation 8). She is also supplied with a certain amount of indoor and full outdoor uniform, and has her washing free.

Before an applicant can be accepted for three years' training she must serve for a probationary period not exceeding three months in the wards, and during this period the matron may at any time terminate her engagement. She will be paid at the rate of £8 per annum during the probationary period.

Having passed the medical officer and dentist, and having completed the probationary period, the applicant will be required to sign an agreement binding herself to three years' service, and undertaking to conform to the rules and regulations of the hospital. The agreement will be dated from the day on which she began to work in the wards.

During the first year a probationer must attend lectures and classes on nursing, and on medicine and surgery as applied to nursing. During her second year she must attend classes for instruction in pharmacy and dispensing. She shall submit herself in due course for examination in nursing, medicine, surgery, and dispensing; and should she fail to pass the examination in any of these subjects, she will, except by special permission of the matron, cease to be a probationer.

The matron may at any time, with the sanction of the treasurer and superintendent, terminate the engagement of a probationer. In such case the probationer will be entitled to receive her salary up to the date of such termination.

If a probationer complete her three years' term, reckoned from the date of her agreement, she shall receive a certificate to that effect signed by the treasurer, superintendent, and matron.

If for any reason whatever a probationer fail to complete her three years' engagement, she shall forfeit a quarter's salary. Under no circumstances will a certificate be given to a probationer who shall not have served her full term of three years.

A probationer whose work or conduct is unsatisfactory, or who fails to pass the examinations mentioned in Regulation 4, may be required to serve for the whole or a part of the last two years of her training at a salary of £8 a year

In case of sickness of more than one week's duration the applicant (referred to in Rule 2 hereof) and the probationer (referred to in the other rules hereof) shall be entitled to no pay from the hospital after the seventh day of sickness; during her sickness she will receive from the proper authority the sickness benefit to which she may be entitled under the National Insurance Act, 1911.

Outdoor uniform (bonnet, cloak, and grey dress) is provided. No nurse or probationer is at any time allowed to wear her print dress or apron out of doors, and the hospital uniform is not to be in any way added to or altered. The wearing of outdoor uniform is optional, but no other than the Guy's nurses' uniform is to be worn. A probationer or nurse may not wear any article of jewellery with her uniform

All probationers are required to join the Nurses' League, the subscription to which is as follows: First year, 5s.; second year, 10s.; third year, 12s.

### Time-table for Day Duty.

| | | | |
|---|---|---|---|
| Called | ... 6.30 A.M. | Tea | ... 5.0 or 5.30 P.M. |
| Breakfast | ... 7.15 A.M. | In wards | ... 5.30 or 6.0 P.M. |
| Prayers | ... 7.45 A.M. | Supper | ... 9.0 or 9.30 P.M. |
| In wards | ... 8.0 A.M. | Prayers | ... 9.30 P.M. |
| Dinner | ... 11.30 or 12.15 | In rooms | ... 10.0 P.M. |
| In wards | ... 12.15 or 1.0 P.M. | | |

### Probationers' Daily Duties.

Probationers are called at half-past six o'clock in the morning, at a quarter-past seven breakfast is served in the dining-hall, at a quarter to eight prayers are said in the chapel, and at eight o'clock they come on duty in the wards. During the morning they clean lamps, inkstands, spatulas, etc.; thoroughly dust the ward; wash lockers and doctor's table; wash window-sills; prepare and serve the patients' luncheon; clear luncheon things; help the nurse with the patients when and as required, and assist with the patients' dinner. At half-past eleven or at a quarter past twelve o'clock, as arranged by the sister, probationers go to dinner, returning to their wards in three-quarters of an hour.

In the afternoons the probationers on duty clear away dinner things, make beds, sweep and dust the ward, and assist with the patients' tea. At five or at half-past five o'clock they take tea in the dining-hall, and half an hour later they return to their wards. During the evening they make beds; wash backs; take round supper; help the nurse when and as required; clean urine-glasses, test-tubes, spitting-cups, etc.; tidy hearths; make up fires for the night, and see that the ward, bathroom, lavatory, etc., are left quite tidy for the night. Probationers have supper at nine o'clock, at half-past nine they go to prayers, which are not compulsory, and after prayers they retire to their rooms. At a quarter to eleven, when the lights are put out, they must be in bed.

(*To be continued.*)

